# GSDB: a database of 3D chromosome and genome structures reconstructed from Hi-C data

**DOI:** 10.1186/s12860-020-00304-y

**Published:** 2020-08-05

**Authors:** Oluwatosin Oluwadare, Max Highsmith, Douglass Turner, Erez Lieberman-Aiden, Jianlin Cheng

**Affiliations:** 1 Department of Computer Science, University of Colorado, Colorado Springs, CO 80918 USA; 2 Department of Electrical Engineering and Computer Science, University of Missouri, Columbia, MO 65211 USA; 3 Elastic Image Software LLC, 21 Walnut Street, Lexington, MA 02421 USA; 4 Department of Genetics, Baylor College of Medicine, Houston, TX 77030 USA

**Keywords:** 3C, Hi-C, 3D chromosome structures, 3D genome structures, GSDB, Genomics, Database

## Abstract

Advances in the study of chromosome conformation capture technologies, such as Hi-C technique - capable of capturing chromosomal interactions in a genome-wide scale - have led to the development of three-dimensional chromosome and genome structure reconstruction methods from Hi-C data. The three dimensional genome structure is important because it plays a role in a variety of important biological activities such as DNA replication, gene regulation, genome interaction, and gene expression. In recent years, numerous Hi-C datasets have been generated, and likewise, a number of genome structure construction algorithms have been developed.

In this work, we outline the construction of a novel Genome Structure Database (GSDB) to create a comprehensive repository that contains 3D structures for Hi-C datasets constructed by a variety of 3D structure reconstruction tools. The GSDB contains over 50,000 structures from 12 state-of-the-art Hi-C data structure prediction algorithms for 32 Hi-C datasets.

GSDB functions as a centralized collection of genome structures which will enable the exploration of the dynamic architectures of chromosomes and genomes for biomedical research. GSDB is accessible at http://sysbio.rnet.missouri.edu/3dgenome/GSDB

## Background

The three-dimensional (3D) organization of the genome plays a significant role in many diverse biological functions and processes including gene expression [1], regulation [2, 3] and transcriptional regulation [4]. Several studies of the architecture of the genome in the cell have linked genome structure to the mechanism of these functions; hence, it is essential to understand the spatial arrangement within the cell nucleus in order to fully elucidate this relation [5–7]. Early studies of the structure of the genome have relied on the use of microscopy techniques such as fluorescence in situ hybridization (FISH), a technique that employs fluorescence probes to detect the presence of a specific chromosome region and the proximity between two regions in a genome sequence [8–10]. Other microscopy methods developed to study the genome organization include stimulated emission depletion (STED) [11], stochastic optical reconstruction microscopy (STORM) [12], and photo-activated localization microscopy (PALM or FPALM) [13, 14]. While these techniques have proven very useful in providing insights into the organization of the genome for DNA fragments or chromatin regions, they are limited and unsuitable for an overall view of the genome-wide inter-and intra-chromosomal relationship study of the genome within the cell nucleus [15].

In order to capture these inter- and intra- chromosomal interactions, a variety of next-generation, high-throughput sequencing technologies have emerged including: 3C [16], 4C [17], 5C [18], Hi-C [19], TCC [20] and ChIA-PET [21, 22]. Out of all these techniques, the Hi-C technique has seen a particularly high usage because of its ability to comprehensively map the chromatin interactions at a genome wide scale.

A Hi-C experiment results in the generation of an interaction frequency (IF) matrix for chromosomal regions (loci) within a chromosome or between any two chromosomes in a population of cells [19, 23–25]. With the advancement of the Hi-C research, sophisticated tools such as GenomeFlow [23], Juicer [26], and HiC-Pro [27] have been developed to generate IF matrices from raw sequence pair reads data [28]. Some methods represent the contact matrix in a sparse 3-column format where columns 1–2 denote the interacting loci and column 3 denotes the number of interactions (or contacts) between the corresponding loci in a Hi-C dataset [24, 29, 30].

Many methods have been developed for chromosome 3D structure reconstruction from chromosome conformation capture (3C) such as the Hi-C data. Generally, these data-driven methods can be grouped into three classes [31] based on how the IF is used for 3D structure construction: distance-based, contact-based and probability-based. First, distance-based methods implement the 3D structure construction through a two-step process.

These methods convert the IF matrix to a distance matrix between loci based on an inverse relation observed from FISH 3D distance data [19]. An optimization function is thereafter used to infer a 3D structure from an initial random structure with the objective of satisfying the distances in the distance matrix as much as possible [6, 24, 29, 32–39]. Second, contact-based methods consider each chromosomal contact as a restraint and apply an optimization algorithm to ensure that the number of contacts in the input contact matrix is satisfied in the 3D structure [30, 40–42]. Third, probability-based methods define a probability measure over the IF, by constructing the structure inference problem as a maximum likelihood problem and thereafter using a sampling e.g. Markov chain Monte Carlo (MCMC) or optimization algorithm to solve the prediction problem [25, 43–45]. Despite the significant progress in the methodological development in 3D chromosome and genome structure modeling and availability of a lot of Hi-C datasets, there is still no public database to store 3D chromosome and genome models for the biological community to use.

Here, we present Genome Structure Database (GSDB), a novel database that contains the chromosome/genome 3D structural models of publicly and commonly used Hi-C datasets reconstructed by twelve state-of-the-art 3D structure reconstruction algorithms at various Hi-C data resolution ranging from 25 KB – 10 MB. The database is organized such that users can view the structures online and download the 3D structures constructed for each dataset by all the reconstruction methods. Our database is the first of its kind to provide a repository of 3D structures and the evaluation results for 3D structures constructed from many Hi-C datasets by different Hi-C data reconstruction methods all in one place.

## Construction and content

### Datasets and normalization

Our Hi-C data is pulled from a variety of sources which we list here. Some datasets were downloaded from the Gene Expression Omnibus (GEO) database, including the Hi-C contact matrices datasets (GEO accession Number: GSE63525) of cell line GM12878 from Rao et al. [46], normalized interaction matrices for each of the four cell types - mouse ES cell, mouse cortex, human ES cell (H1), and IMR90 fibroblasts – (GEO accession Number: GSE35156) [47, 48], and the Hi-C contact matrices datasets (GEO Accession Number: GSE18199) of karyotypically normal human lymphoblastic cell line (GM06990, K562) [19]. All other Hi-C datasets were obtained from the ENCODE project repository [49], and the GEO accession Number and the ENCODE ID for each dataset are available on the GSDB website. Currently, this GSDB contains over 50,000 structural models of various resolutions reconstructed from 32 unique Hi-C datasets by 12 state-of-the-art 3D genome/chromosome modeling methods. More Hi-C datasets will be used to build 3D models as they are available. Hi-C data normalization is an important process in 3D structure reconstruction from Hi-C data, because the raw contact count matrix obtained from 3C experiments may contain numerous systematic biases, such as GC content, length of restriction fragments, and other technical biases that could influence the 3D structure reconstruction [50–54]. Consequently, all the contact matrices were normalized prior to applying the 3D structur reconstruction algorithms. Contact matrices from Dixon et al. [47] were obtained with Yaffey-Tanay normalization already applied. All other contact matrices were normalized using the vanilla coverage method as described in Rao et al. [46].

### Database implementation

The GSDB website interface was implemented using HTML, PHP and JavaScript, and the database was implemented in MySQL (https://www.mysql.com/) . The online 3D structure visualization was done through 3Dmol viewer, a molecular visualization JavaScript library [55].

### 3D modeling algorithms included

We used twelve existing algorithms for the 3D structure construction. We selected a mixture of distance-based, contact-based, and probability-based algorithms [31]. We first describe the distance-based algorithms. LorDG [24] uses a nonlinear Lorentzian function as the objective function with the main objective of maximizing the satisfaction of realistic restraints rather than outliers. LorDG uses a gradient ascent algorithm to optimize the objective function. 3DMax [29] used a maximum likelihood approach to infer the 3D structures of a chromosome from Hi-C data. A log-likelihood was defined over the objective function which was maximized through a stochastic gradient ascent algorithm with per-parameter learning rate [56]. Chromosome3D [32] uses distance geometry simulated annealing (*DGSA*) to construct chromosome 3D structure by translating the distance to positions of the points representing loci. Chromosome3D adopts the Crystallography & NMR System (CNS) suite [57] which has been rigorously tested for protein structure construction for the 3D genome structure prediction from Hi-C data. HSA [6] introduced an algorithm capable of taking multiple contact matrices as input to improve performance. HSA can generate same structure irrespective of the restriction enzyme used in the Hi-C experiment. ChromSDE [37] (Chromosome Semi-Definite Embedding) framed the 3D structure reconstruction problem as a semi-definite programming problem. Shrec3D [38] formulated the 3D structure reconstruction problem *as* a graph problem and attempts to find the shortest-path distance between two nodes on the graph. The length of a link is determined as the inverse contact frequency between its end nodes. Each fragment is regarded as the nodes connected by a link. The represented 3D structure for a Hi-C data is one in which distance between the nodes is the shortest. InfoMod3DGen [39] converts the IF to a distance matrix and used an expectation-maximization (EM) based algorithm to infer the 3D structure.

In the contact-based category, we used MOGEN [30] and GEM [41] for the 3D structure reconstruction. MOGEN [30] does not require the conversion of IF to distances and is suitable for large-scale genome structure modeling. GEM [41] considers both Hi-C data and conformational energy derived from knowledge about biophysical models for 3D structure modeling. It used a manifold learning framework, which is aimed at extracting information embedded within a high-dimensional space, in this case the Hi-C data.

Lastly, in the probability-based category, Pastis [25] defined a probabilistic model of IF and casted the 3D inference problem as a maximum likelihood problem. It defined a Poisson model to fit contact data and used an optimization algorithm to solve it. SIMDA3D [45] used a Bayesian approach to infer 3D structures of chromosomes from single cell Hi-C data.

### Computational model reconstruction

The GSDB chromosome structure generation was done on three server machines: a x86_64 bit Redhat-Linux server consisting of multi-core Intel(R) Xeon(R) CPU E7-L8867 @ 2.13GHz with 120 GB RAM, x86_64 bit Redhat-Linux server consisting of multi-core Intel(R) Xeon(R) CPU E5649 @ 2.53GHz with 11GB RAM, x86_64 bit Redhat-Linux server consisting of multi-core AMD Opteron (tm) Processor 4284 @ 3.0GHz with 62GB RAM, and a high-performance computing cluster (Lewis) with Linux. Using a high-performance computing (HPC) cluster machine, we allocated 10 cores, 80G of memory, with a time limit of 2 days for each chromosome structure reconstruction task per algorithm. Structures not constructed within 48 h were terminated.

## Utility and discussion

All the 3D structures in the GSDB have been pre-generated, so that the 3D structure visualization is faster and can be easily downloaded. The steps to navigating the database have been separated into five sections as follows:

### Browse the database

**Fig. 1:**
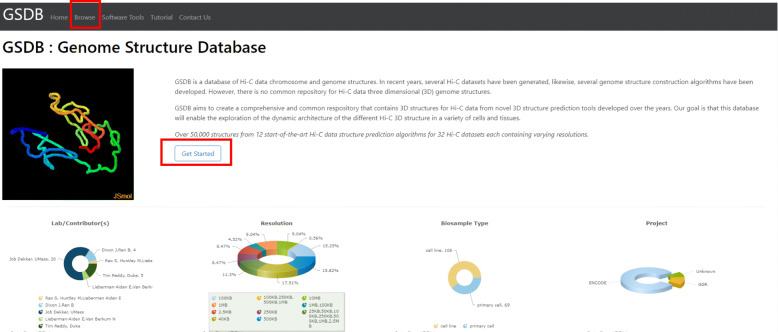
Browse the database Highlights the two ways to access the database from the homepage. Clicking on the “Browse” menu in the Navigation tab or on the “Get started” button on the home page will load the Database search window

Click on “Browse” menu in the navigation bar to load the full list of the Hi-C datasets. Alternatively, users can click on the “Get Started” button on the homepage (Fig. 1).

### Search the database

**Fig. 2:**
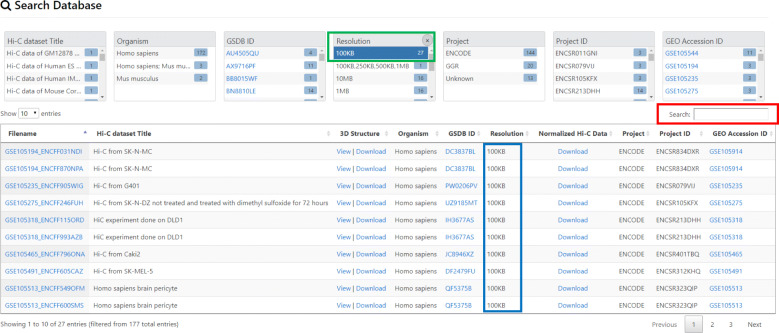
Database search and display An example of data search using the two approaches for searching. First, search by clicking on an item on the “Summary Pane” highlighted in green. The figure shows when the user clicks on Resolution 100 kb, all the datasets with 100 kb resolutions are listed. Second, the user can search by typing the key word in the “Search Pane” highlighted in red

The GSDB provides two ways to search for a Hi-C data and its corresponding 3D models:

- GSDB provides a summary of the information provided in the database through a Summary Pane. By clicking on a property/item in the summary, the user can search the database for all the Hi-C data containing this property and their corresponding 3D structural models. (Fig. 2)
- Users can search the database by typing the keywords about the filename, title of Hi-C data, Hi-C data resolution, project that Hi-C data was generated from (e.g. ENCODE), project ID, and the GEO accession No in the “Search Pane” (Fig. 2).

### Download

**Fig. 3:**
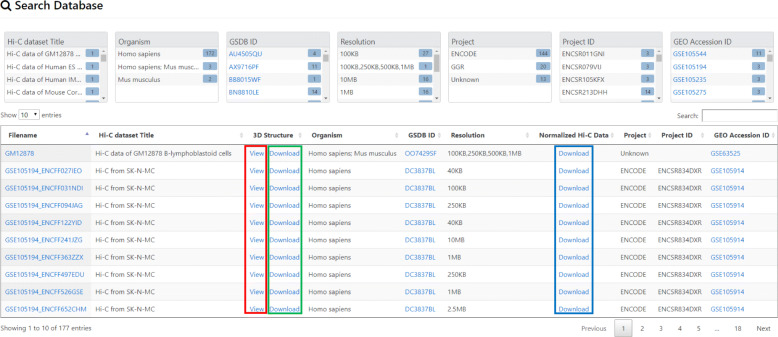
3D structure display and download In the “3D Structure” column, highlighted in red is the “View” link to display the 3D structure for a Hi-C data. Highlighted in green is the “Download” link to download the 3D structures constructed by the different algorithms for the Hi-C data. Pressing on the “Download” link will download the 3D structures for all the algorithms for a Hi-C data. In the “Normalized Hi-C Data” column, the “Download” link is highlighted in blue. Pressing on the “Download” link will download the Normalized Hi-C data used for 3D structure construction

Users can download the 3D structures by clicking on the “Download” link in the “3D Structure Column” (Fig. 3). The normalized Hi-C data used for the 3D structure generation for all the algorithms can also be downloaded by clicking on the “Download” link in the “Normalized Hi-C Data” column (Fig. 3). Structures may be downloaded in PDB, G.PDB and Spacewalk format.

#### File formats

The current de facto standard for representation of three dimensional chromosomal structures is the (Protein Data Bank) PDB file format where genomic bins are represented as ATOM lines. However, this format has disadvantages as it excludes other useful pieces of information such as: The reference genome used in alignment, the cell line, the chromosome being represented and the genomic coordinates corresponding to the displayed bins. Consequently we introduce the G.PDB file format which includes this information through the insertion of a HEADER line as well as REMARK lines following each ATOM line. G.PDB files are usable within all existing visualization tools which utilize standard PDB files. In addition to the G.PDB file we represent structure using the .sw (spacewalk) format, so that structures can be visualized using the SpaceWalk tool [58].

### 3D structure and Heatmap visualization

**Fig. 4:**
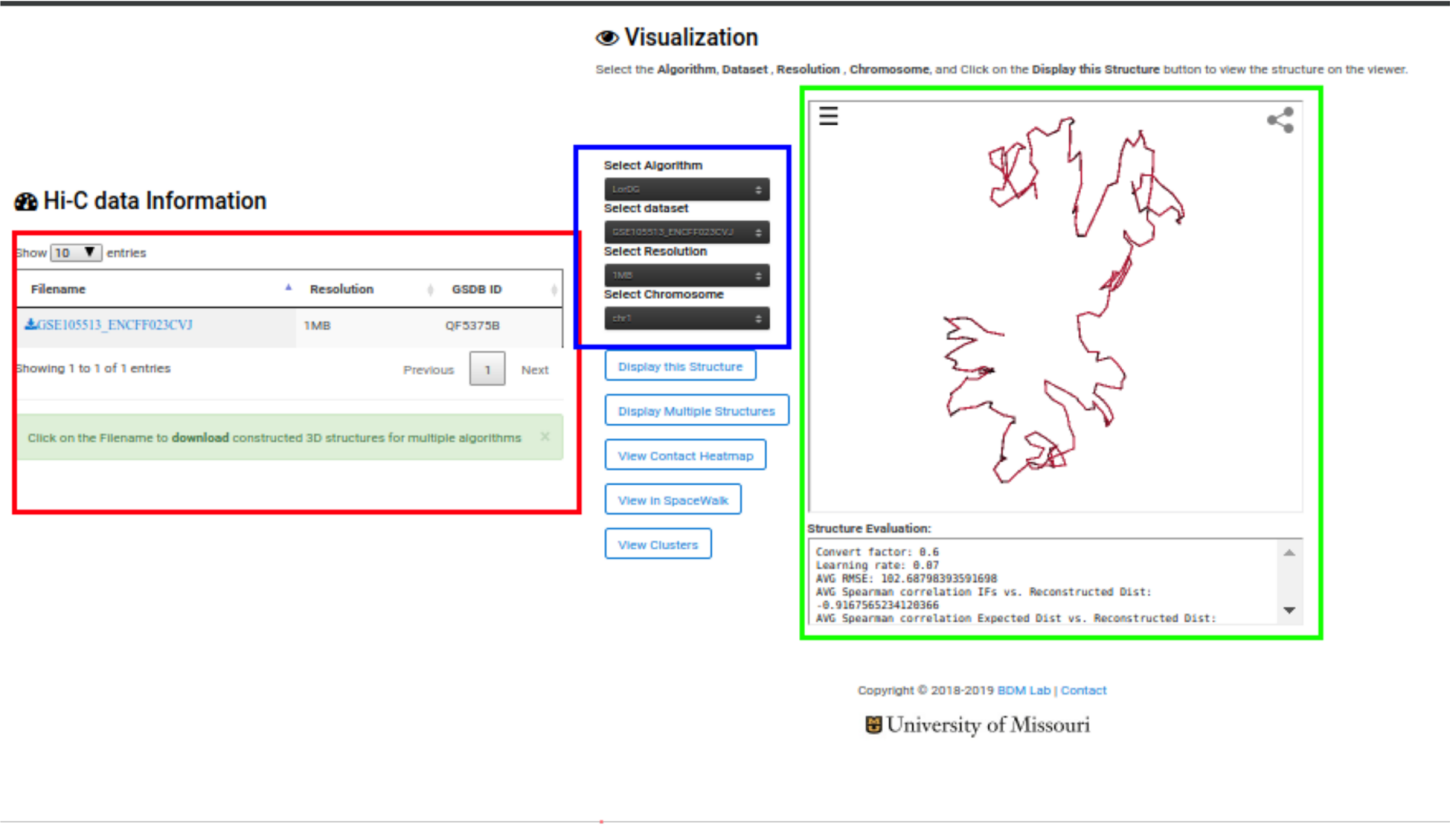
Data visualization The figure shows the output displayed when a user clicks on the “View link” for the GM12878 dataset. The red highlighted section shows the information about the Resolution(s) available for the Hi-C data. The blue highlighted section displays the structure available for the Hi-C data. The green highlighted section shows the evaluation result available for the Hi-C data. It displays the Spearman Correlation between the output structure and the input Hi-C data, and other evaluation result obtained. To evaluate each 3D structure, we compute the distance Spearman’s correlation coefficient (dSCC) between reconstructed distances and distances obtained from the Hi-C datasets. The value of dSCC is in the range of − 1 to + 1, where a higher value is better. For distance-based methods, we report the conversion factor (α) used for the IF to distance conversion. For LorDG and 3DMax, which use gradient ascent optimization algorithm, we report the learning rate used for the optimization process. The parameters used by each method to generate 3D structures are available on GSDB GitHub page

**Fig. 5:**
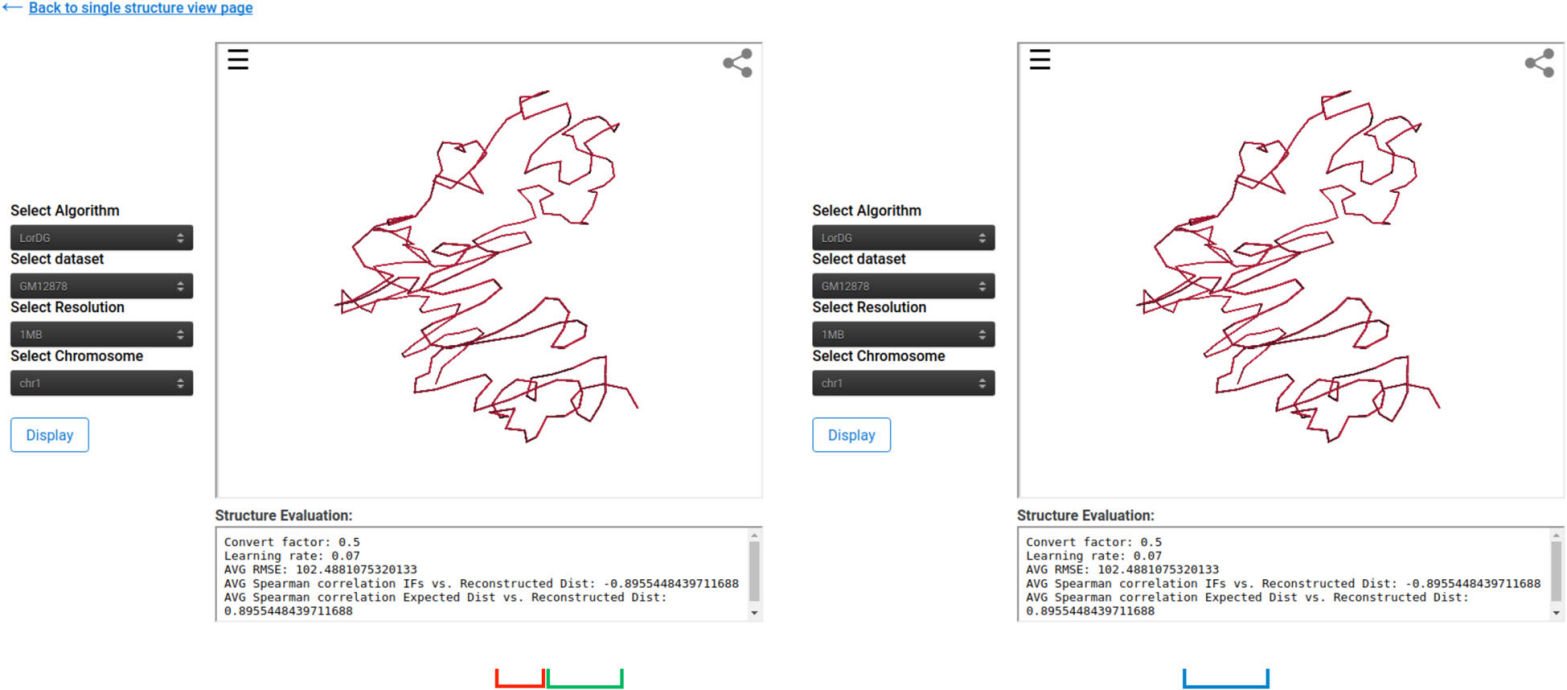
Multiple 3D structure visualization The figures shows the output displayed when a user clicks the “Display Multiple Structures” button. The multiple structure view permits the comparison of structures using different 3D structure algorithms or different Hi-C contact matrices

To view the details and structures for a Hi-C data, click on the “View” link in the “3D Structure Column” (Fig. 3). The data information and visualization tab will be displayed (Fig. 4). To show the 3D structure, select the algorithm, dataset, chromosome, and press “Display this Structure” button. The structure will be displayed on the viewer. The modeling parameters and the reconstruction quality (e.g. the Spearman’s correlation between reconstructed distances and expected distances) are reported in the box under the viewer. To compare two structures at the same time, press the “Display Multiple Structures” button. Two structures will be displayed side by side with two distinct options for selecting each visualization’s 3D structure algorithm and dataset (Fig. 5). To view a heatmap of the 2D contact matrix used to reconstruct the 3D structure, click the “View Contact Heatmap” button.

**Fig. 6:**
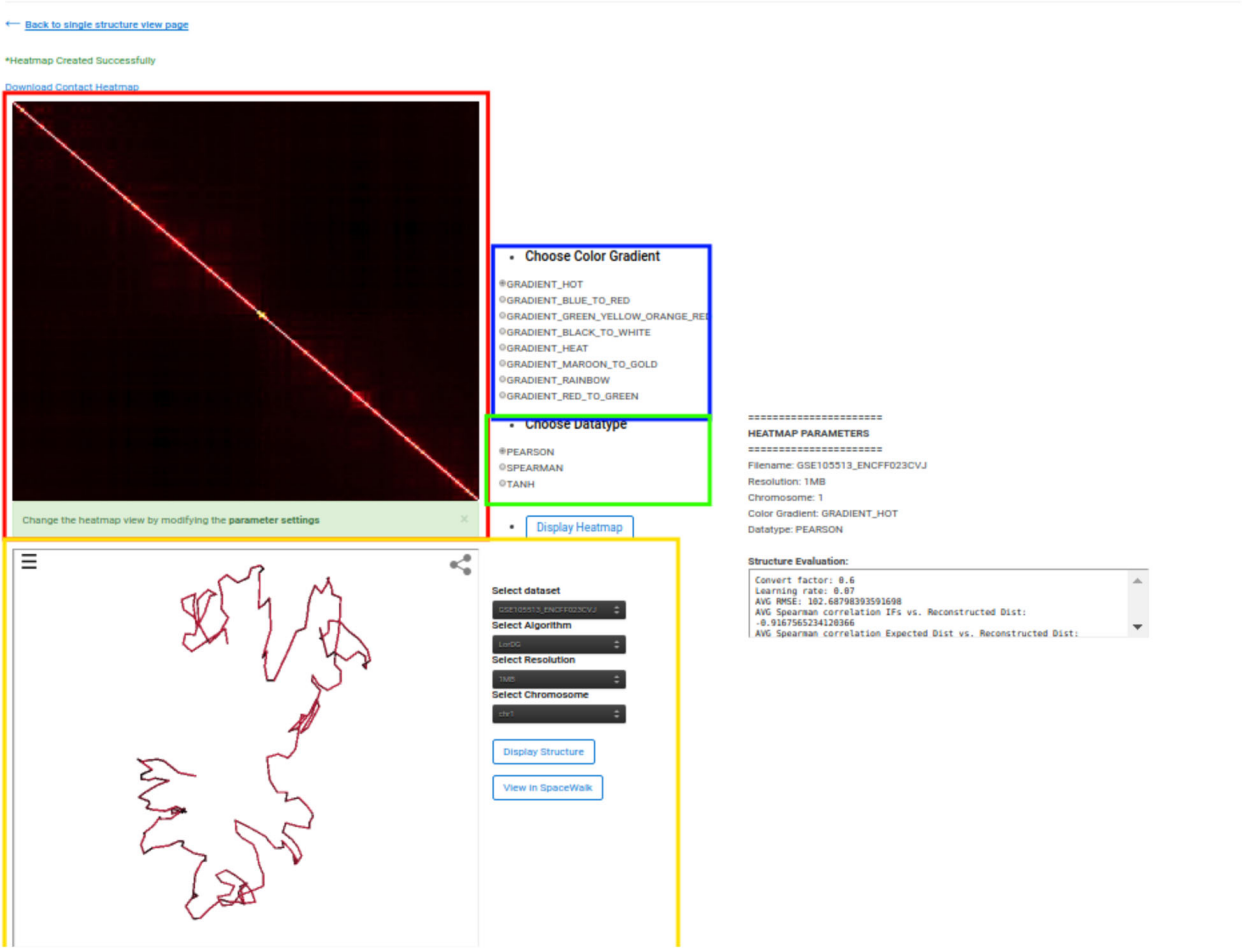
Heatmap visualization The figure shows the output displayed when a user clicks the yellow outlined “View Contact Heatmap” button shown in Fig. 4. The figure highlighted in red indicates heat map visualization of the selected 2D chromosomal contact map. The radio buttons outlined in blue display options for the heat map colour. The radio buttons outlined in green indicate functions that may be applied to the raw contact matrix prior to heat map construction so as toimprove visualization. The window outlined in yellow displays a visualization of the 3D structure

The heat map can be configured with a variety of helper visualization functions as well as color settings to customize visualization (Fig. 6). To view the structure in the external tool spacewalk [58] press “View in Spacewalk”. The user will be redirected to the spacewalk website where model can be loaded with the corresponding URL.

### Evaluation of structure

**Fig. 7:**
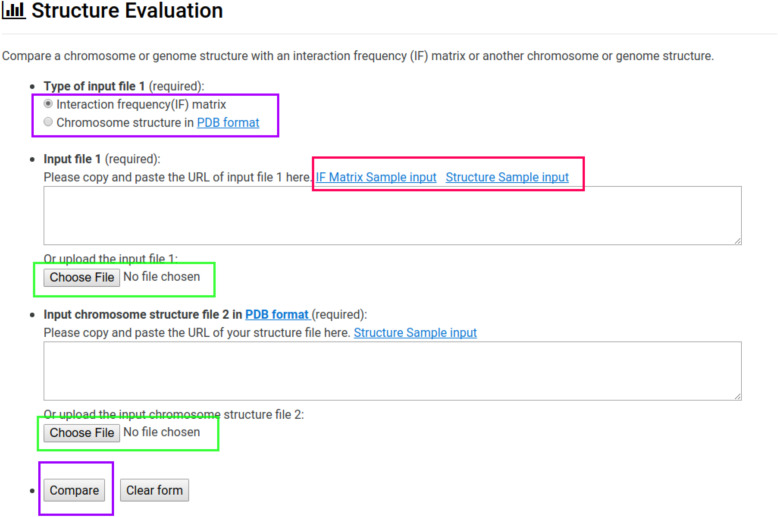
Evaluation The figure shows the window displayed if a user selects the “Evaluation” tab. The purple box displays the radio buttons which determine whether a comparison will involve 2 structures stored in the Protein Data Bank (PDB) format or a structure in the PDB format and an IF matrix. The green boxes indicate buttons for selecting the files to be compared. The red box denotes links to sample data for testing comparison. The purple box indicates the evaluation button, which will submit the comparison job

The GSDB contains an evaluation module which permits users to evaluate their own 3D models by comparing model distances to the expected distances of an IF matrix or another 3D model (Fig. 7). Upon uploading two PDB files or a PDB file and an IF matrix file and clicking on “Compare” button, users are provided with a collection of evaluation scores including: Spearman Correlation, Pearson Correlation and Root Mean Squared Distance (RMSD). Users may also load G.PDB files wherever PDB files are accepted.

**Fig. 8:**
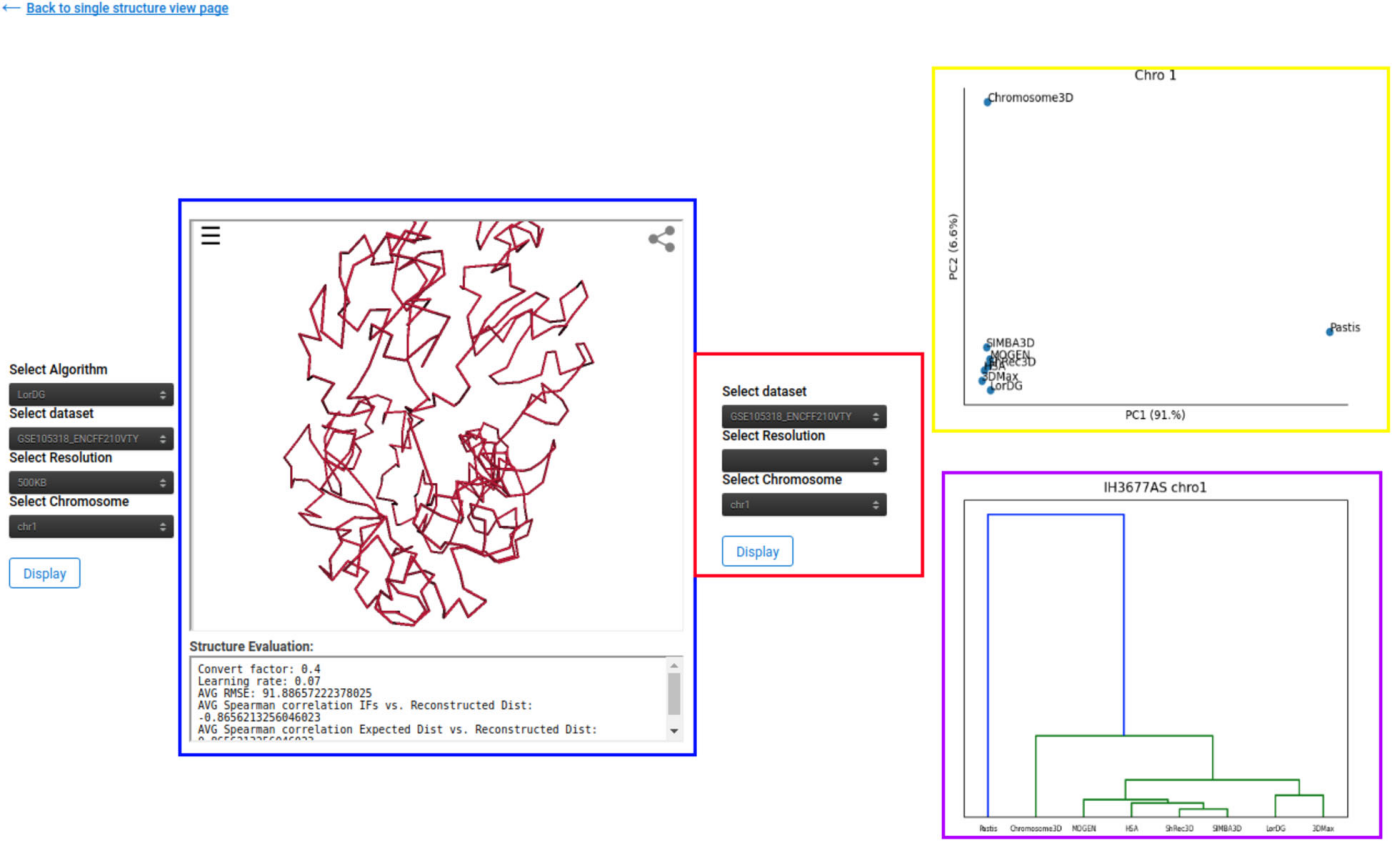
Evaluation The figure shows the window displayed if the user selects the cluster Button from Fig. 4. The contents of the Yellow box display a 2D representation of the principle component analysis (PCA) values of each structure selected using the parameters contained in the red box. The contents of the purple box display hierarchical agglomerative clustering of structures

### Tool selection

Because the ground truth structure of the 3D genome has not been holistically validated, determination of which 3D structure predicting algorithm is best remains an unsolved problem. GSDB provides users with guidance in tool selection by including a cluster page. This page displays unsupervised principal component analysis and hierarchical agglomerative clustering of the structures predicted by different tools (Fig. 8). Certain tools remove low coverage bins in the 3D structure generation consequently we only include structures with the same number of points in all unsupervised comparisons.

## Conclusions

The GSDB contains 3D structures generated from different Hi-C structure reconstruction algorithms for Hi-C data collected from multiple sources. To the best of our knowledge, it is the first repository for 3D structures generated from multiple Hi-C reconstruction algorithms. Currently, our database contains over 50,000 structures reconstructed for 32 Hi-C datasets by 11 modeling algorithms. The normalized Hi-C dataset used and 3D structures generated from all the algorithms are available to be downloaded. This database will enable the fast and easy exploration of the dynamic architecture of the different Hi-C 3D structure in a variety of cells to improve our understanding of the structural organization of various organisms’ chromosome and genome 3D structures. In addition, we envision that it will be helpful to researchers and scientist to keep track of the performance of the existing approaches for 3D structure construction, and also lead to the development of novel methods that outperform existing approaches. Future directions of the GSDB will include the integration of more algorithms and latest Hi-C datasets generated as the research in 3D structure construction expands.

## Data Availability

GSDB database is freely available at the URL http://sysbio.rnet.missouri.edu/3dgenome/GSDB. Scripts and the parameters used for the 3D structure generation for each algorithm are available at https://github.com/BDM-Lab/GSDB

## Abbreviations

3D: Three Dimensional
MCMC: Markov Chain Monte Carlo
GSDB: Genome Structure Database
*DGSA*: *Distance Geometry Simulated Annealing*
FISH: Fluorescen tIn Situ Hybridization
STED: stimulated emission depletion
STORM: stochastic optical reconstruction microscopy
PALM: photo-activated localization microscopy
RMSD: root mean squared distance
PDB: Protein Database
GEO: Gene Expression Omnibus
HPC: High Performance Computing

## References

[1] de Laat W, Grosveld F (2003). Spatial organization of gene expression: the active chromatin hub. Chromosom Res.

[2] Dekker J (2008). Gene regulation in the third dimension. Science.

[3] Dekker J, Marti-Renom MA, Mirny LA (2013). Exploring the three-dimensional organization of genomes: interpreting chromatin interaction data. Nat Rev Genet.

[4] Miele A, Dekker J (2008). Long-range chromosomal interactions and gene regulation. Mol BioSyst.

[5] de Wit E, De Laat W (2012). A decade of 3C technologies: insights into nuclear organization. Genes Dev.

[6] Zou C, Zhang Y, Ouyang Z (2016). HSA: integrating multi-track hi- C data for genome-scale reconstruction of 3D chromatin structure. Genome Biol.

[7] Park J, Lin S (2016). Impact of data resolution on three-dimensional structure inference methods. BMC Bioinformatics.

[8] Amann R, Fuchs BM (2008). Single-cell identification in microbial communities by improved fluorescence in situ hybridization techniques. Nat Rev Microbiol.

[9] Langer-Safer PR, Levine M, Ward DC (1982). Immunological method for mapping genes on Drosophila polytene chromosomes. Proc Natl Acad Sci.

[10] Cremer T, Cremer C (2001). Chromosome territories, nuclear architecture and gene regulation in mammalian cells. Nat Rev Genet.

[11] Westphal V, Rizzoli SO, Lauterbach MA, Kamin D, Jahn R, Hell SW (2008). Video-rate far-field optical nanoscopy dissects synaptic vesicle movement. Science.

[12] Rust MJ, Bates M, Zhuang X (2006). Sub-diffraction-limit imaging by stochastic optical reconstruction microscopy (STORM). Nat Methods.

[13] Betzig E, Patterson GH, Sougrat R, Lindwasser OW, Olenych S, Bonifacino JS, Davidson MW, Lippincott-Schwartz J, Hess HF (2006). Imaging intracellular fluorescent proteins at nanometer resolution. Science.

[14] Huang B, Babcock H, Zhuang X (2010). Breaking the diffraction barrier: super-resolution imaging of cells. Cell.

[15] Williamson I, Berlivet S, Eskeland R, Boyle S, Illingworth RS, Paquette D, Dostie J, Bickmore WA (2014). Spatial genome organization: contrasting views from chromosome conformation capture and fluorescence in situ hybridization. Genes Dev.

[16] Dekker J, Rippe K, Dekker M, Kleckner N (2002). Capturing chromosome conformation. Science.

[17] Simonis M, Klous P, Splinter E, Moshkin Y, Willemsen R, De Wit E, Van Steensel B, De Laat W (2006). Nuclear organization of active and inactive chromatin domains uncovered by chromosome conformation capture–on-chip (4C). Nat Genet.

[18] Dostie J, Richmond TA, Arnaout RA, Selzer RR, Lee WL, Honan TA, Rubio ED, Krumm A, Lamb J, Nusbaum C, Green RD (2006). Chromosome conformation capture carbon copy (5C): a massively parallel solution for mapping interactions between genomic elements. Genome Res.

[19] Lieberman-Aiden E, Van Berkum NL, Williams L, Imakaev M, Ragoczy T, Telling A, Amit I, Lajoie BR, Sabo PJ, Dorschner MO, Sandstrom R (2009). Comprehensive mapping of long-range interactions reveals folding principles of the human genome. Science.

[20] Kalhor R, Tjong H, Jayathilaka N, Alber F, Chen L (2012). Genome architectures revealed by tethered chromosome conformation capture and population-based modeling. Nat Biotechnol.

[21] Fullwood MJ, Liu MH, Pan YF, Liu J, Xu H, Mohamed YB, Orlov YL, Velkov S, Ho A, Mei PH, Chew EG (2009). An oestrogen- receptor-α-bound human chromatin interactome. Nature.

[22] Li G, Fullwood MJ, Xu H, Mulawadi FH, Velkov S, Vega V, Ariyaratne PN, Mohamed YB, Ooi HS, Tennakoon C, Wei CL (2010). ChIA-PET tool for comprehensive chromatin interaction analysis with paired-end tag sequencing. Genome Biol.

[23] Trieu T, Oluwadare O, Wopata J, Cheng J. GenomeFlow: a comprehensive graphical tool for modeling and analyzing 3D genome structure. Bioinformatics. 2018;35(8):1416–18. 10.1093/bioinformatics/bty802 PMC6477968 30215673

[24] Trieu T, Cheng J (2016). 3D genome structure modeling by Lorentzian objective function. Nucleic Acids Res.

[25] Varoquaux N, Ay F, Noble WS, Vert JP (2014). A statistical approach for inferring the 3D structure of the genome. Bioinformatics.

[26] Durand NC, Shamim MS, Machol I, Rao SS, Huntley MH, Lander ES, Aiden EL (2016). Juicer provides a one-click system for analyzing loop-resolution hi-C experiments. Cell Syst.

[27] Servant N, Varoquaux N, Lajoie BR, Viara E, Chen CJ, Vert JP, Heard E, Dekker J, Barillot E (2015). HiC-pro: an optimized and flexible pipeline for hi-C data processing. Genome Biol.

[28] Ay F, Noble WS (2015). Analysis methods for studying the 3D architecture of the genome. Genome Biol.

[29] Oluwadare O, Zhang Y, Cheng J (2018). A maximum likelihood algorithm for reconstructing 3D structures of human chromosomes from chromosomal contact data. BMC Genomics.

[30] Trieu T, Cheng J (2015). MOGEN: a tool for reconstructing 3D models of genomes from chromosomal conformation capturing data. Bioinformatics.

[31] Oluwadare O, Highsmith M, Cheng J. An overview of methods for reconstructing 3-D chromosome and genome structures from hi-C data. Biol Proced Online. 2019. 10.1186/s12575-019-0094-0. 10.1186/s12575-019-0094-0 PMC6482566 31049033

[32] Adhikari B, Trieu T, Cheng J (2016). Chromosome3D: reconstructing three-dimensional chromosomal structures from hi-C interaction frequency data using distance geometry simulated annealing. BMC Genomics.

[33] Fraser J, Rousseau M, Shenker S, Ferraiuolo MA, Hayashizaki Y, Blanchette M, Dostie J (2009). Chromatin conformation signatures of cellular differentiation. Genome Biol.

[34] Hua K, Ma B. EVR: reconstruction of bacterial chromosome 3D structure models using error-vector resultant algorithm. BMC Genomics. 2019;20:738. 10.1186/s12864-019-6096-0. 10.1186/s12864-019-6096-0 PMC6794827 31615397

[35] Szalaj P, Michalski PJ, Wróblewski P, Tang Z, Kadlof M, Mazzocco G, Ruan Y, Plewczynski D (2016). 3D-GNOME: an integrated web service for structural modeling of the 3D genome. Nucleic Acids Res.

[36] Rieber L, Mahony S (2017). miniMDS: 3D structural inference from high-resolution hi-C data. Bioinformatics.

[37] Zhang Z, Li G, Toh KC, Sung WK (2013). Inference of spatial organizations of chromosomes using semi-definite embedding approach and Hi-C data. Annual international conference on research in computational molecular biology.

[38] Lesne A, Riposo J, Roger P, Cournac A, Mozziconacci J (2014). 3D genome reconstruction from chromosomal contacts. Nat Methods.

[39] Wang S, Xu J, Zeng J (2015). Inferential modeling of 3D chromatin structure. Nucleic Acids Res.

[40] Nowotny J, Ahmed S, Xu L, Oluwadare O, Chen H, Hensley N, Trieu T, Cao R, Cheng J (2015). Iterative reconstruction of three- dimensional models of human chromosomes from chromosomal contact data. BMC Bioinformatics.

[41] Zhu G, Deng W, Hu H, Ma R, Zhang S, Yang J (2018). Reconstructing spatial organizations of chromosomes through manifold learning. Nucleic Acids Res.

[42] Paulsen J, Sekelja M, Oldenburg AR, Barateau A, Briand N, Delbarre E, Shah A, Sørensen AL, Vigouroux C, Buendia B, Collas P (2017). Chrom3D: three-dimensional genome modeling from hi-C and nuclear Lamin-genome contacts. Genome Biol.

[43] Hu M, Deng K, Qin Z, Dixon J, Selvaraj S, Fang J, Ren B, Liu JS (2013). Bayesian inference of spatial organizations of chromosomes. PLoS Comput Biol.

[44] Tjong H, Li W, Kalhor R, Dai C, Hao S, Gong K, Zhou Y, Li H, Zhou XJ, Le Gros MA, Larabell CA (2016). Population-based 3D genome structure analysis reveals driving forces in spatial genome organization. Proc Natl Acad Sci.

[45] Rosenthal M, Bryner D, Huffer F, Evans S, Srivastava A, Neretti N. Bayesian estimation of 3D chromosomal structure from single-cell hi-C data. J Comput Biol. 2019;26(11):1191–1202. 10.1089/cmb.2019.0100. 10.1089/cmb.2019.0100 PMC6856950 31211598

[46] Rao SS, Huntley MH, Durand NC, Stamenova EK, Bochkov ID, Robinson JT, Sanborn AL, Machol I, Omer AD, Lander ES, Aiden EL (2014). A 3D map of the human genome at kilobase resolution reveals principles of chromatin looping. Cell.

[47] Dixon JR, Selvaraj S, Yue F, Kim A, Li Y, Shen Y, Hu M, Liu JS, Ren B (2012). Topological domains in mammalian genomes identified by analysis of chromatin interactions. Nature.

[48] GSE35156, Normalized Hi-C data. http://chromosome.sdsc.edu/mouse/hi- c/download.html. Accessed 10 Apr 2019.

[49] ENCODE Project Consortium (2012). An integrated encyclopedia of DNA elements in the human genome. Nature.

[50] Imakaev M, Fudenberg G, McCord RP, Naumova N, Goloborodko A, Lajoie BR, Dekker J, Mirny LA (2012). Iterative correction of hi-C data reveals hallmarks of chromosome organization. Nat Methods.

[51] Hu M, Deng K, Selvaraj S, Qin Z, Ren B, Liu JS (2012). HiCNorm: removing biases in hi-C data via Poisson regression. Bioinformatics.

[52] Knight PA, Ruiz D (2013). A fast algorithm for matrix balancing. IMA J Numer Anal.

[53] Cournac A, Marie-Nelly H, Marbouty M, Koszul R, Mozziconacci J (2012). Normalization of a chromosomal contact map. BMC Genomics.

[54] Yaffe E, Tanay A (2011). Probabilistic modeling of hi-C contact maps eliminates systematic biases to characterize global chromosomal architecture. Nat Genet.

[55] Rego N, Koes D (2014). 3Dmol. Js: molecular visualization with WebGL. Bioinformatics.

[56] Duchi J, Hazan E, Singer Y (2011). Adaptive subgradient methods for online learning and stochastic optimization. J Mach Learn Res.

[57] Brünger AT, Adams PD, Clore GM, DeLano WL, Gros P, Grosse-Kunstleve RW, Jiang JS, Kuszewski J, Nilges M, Pannu NS, Read RJ (1998). Crystallography and NMR system: a new software suite for macromolecular structure determination. Acta Crystallogr D Biol Crystallogr.

[58] Turner D, Spacewalk (2019). GitHub repository.

