# Correction to: GSDB: a database of 3D chromosome and genome structures reconstructed from Hi-C data

**DOI:** 10.1186/s12860-020-00305-x

**Published:** 2020-08-18

**Authors:** Oluwatosin Oluwadare, Max Highsmith, Douglass Turner, Erez Lieberman Aiden, Jianlin Cheng

**Affiliations:** 1 Department of Computer Science, University of Colorado, Colorado Springs, CO 80918 USA; 2 Department of Electrical Engineering and Computer Science, University of Missouri, Columbia, MO 65211 USA; 3 Elastic Image Software LLC, 21 Walnut Street, Lexington, MA 02421 USA; 4 Department of Genetics, Baylor College of Medicine, Houston, TX 77030 USA

**Correction to: BMC Mol and Cell Biol 21, 60 (2020)**

**https://doi.org/10.1186/s12860-020-00304-y**

Following publication of the original article [1], an error was reported in the name of Erez Lieberman Aiden in the author group.

The incorrect author name is:

Erez Lieberman-Aiden

The correct author name is:

Erez Lieberman Aiden

The original article [1] has been corrected.
